# Cost‐minimization analysis of a wearable cardioverter defibrillator in adult patients undergoing ICD explant procedures: Clinical and economic implications

**DOI:** 10.1002/clc.23709

**Published:** 2021-08-24

**Authors:** Giuseppe Boriani, Lorenzo Giovanni Mantovani, Paolo Angelo Cortesi, Roberto De Ponti, Antonio D'Onofrio, Giuseppe Arena, Antonio Curnis, Giovanni Forleo, Federico Guerra, Maurizio Porcu, Giuseppe Sgarito, Giovanni Luca Botto

**Affiliations:** ^1^ Cardiology Division, Department of Biomedical, Metabolic and Neural Sciences University of Modena and Reggio Emilia Policlinico di Modena Italy; ^2^ Research Centre on Public Health (CESP) University of Milano‐Bicocca Monza Italy; ^3^ Value‐Based Healthcare Unit IRCCS Multimedica Sesto San Giovanni Italy; ^4^ Department of Heart and Vessels Ospedale di Circolo‐University of Insubria Varese Italy; ^5^ Cardiology Division – Electrophysiology Department – AORN dei Colli – Ospedale Monaldi Napoli Italy; ^6^ Cardiology Department Azienda Usl Toscana Nord Ovest Massa Carrara Italy; ^7^ Cardiology Department Presidio Ospedaliero di Brescia, ASST Spedali Civili Brescia Italy; ^8^ Cardiology Department, Electrophysiology and Arrhtymology Division Ospedale Luigi Sacco ‐ Polo Universitario Milan Italy; ^9^ Cardiology and Arrhytmology Clinic Azienda Ospedaliero Universitaria Ospedali Riuniti Ancona Italy; ^10^ Cardiology Department Azienda Ospedaliera “G. Brotzu” Cagliari Italy; ^11^ Cardiology Department, Electrophysiology and Arrhtymology Division A.R.N.A.S. Ospedali Civico Palermo Italy; ^12^ Cardiology – Electrophysiology Division, Department of Medicine Ospedale di Circolo Rho, Ospedale Salvini Garbagnate M.se, ASST Rhodense Milan Italy

**Keywords:** cost‐minimization analysis, health technology assessment, ICD explant, sudden cardiac death, ventricular arrhythmia, wearable cardioverter defibrillator

## Abstract

**Aims:**

Patients with permanently increased risk of sudden cardiac death (SCD) can be protected by implantable cardioverter defibrillators (ICD). If an ICD must be removed due to infection, for example, immediate reimplantation might not be possible or indicated. The wearable cardioverter defibrillator (WCD) is an established, safe and effective solution to protect patients from SCD during this high‐risk bridging period. Very few economic evaluations on WCD use are currently available.

**Methods:**

We conducted a systematic review to evaluate the available evidence of WCD in patients undergoing ICD explant/lead extraction. Additionally, a decision model was developed to compare use and costs of the WCD with standard therapy (in‐hospital stay). For this purpose, a cost‐minimization analysis was conducted, and complemented by a one‐way sensitivity analysis.

**Results:**

In the base case scenario, the WCD was less expensive compared to standard therapy. The cost‐minimization analysis showed a cost reduction of €1782 per patient using the WCD. If costs of standard care were changed, cost savings associated with the WCD varied from €3500 to €0, assuming costs for standard care of €6800 to €3600.

**Conclusion:**

After ICD explantation, patients can be safely and effectively protected from SCD after hospital discharge through WCD utilization. Furthermore, the use of a WCD for this patient group is cost saving when compared to standard therapy.

## 1 INTRODUCTION

Implantable cardioverter defibrillator (ICD) use in primary and secondary prevention of sudden cardiac death (SCD) has been the standard of care for many years. Device explantation is necessary if lead or pocket infection, lead fracture or lead malfunctions occur.^1^, ^2^, ^3^ Immediate reimplantation is not always possible or even indicated. Leading national and international cardiology societies recommend ICD reimplantation only after complete eradication of the responsible germ.^4^, ^5^, ^6^, ^7^ Mortality after device removal with simultaneous antibiotic therapy ranges between 8 and 26.9%. If patients are treated with antibiotic therapy alone, this range increases to 31–66%.^8^ Early reimplantation can result in recurrent infection. At the same time, there is a substantial risk of ventricular tachycardia (VT) or ventricular fibrillation (VF) events leading to SCD in these patients, since this patient group is characterized by an established and permanent risk.^9^, ^10^, ^11^ Several weeks of inpatient monitoring would be indicated, but this is neither economically attractive nor reasonable for patients in terms of quality of life due to complications such as thrombosis, nosocomial infection, and psychological stress. At the same time, associated costs are increasing globally, which corresponds to the overall increase in the rates of ICD implantations and multiplying device and lead replacements over the years. Inpatient hospital stay is the standard pathway for patients after ICD extraction.

The incidence of cardiovascular implantable electronic device infections is increasing faster than the device implantation rate. Between 1993 and 2008, an increase in infections ranging from 96 to 210% was reported.^2^, ^8^ Overall, this leads to a considerable burden on healthcare systems. The management of patients waiting for an ICD reimplantation should therefore be individualized, safe and effective, as well as economically sustainable.

The wearable cardioverter defibrillator (WCD) has the potential for being a useful bridging tool to cover the time‐period in which patients are normally unprotected as they wait for their infection to resolve.

The WCD is a noninvasive external defibrillator that continuously records and analyzes ECG sequences. In case potentially lethal VT/VF episodes occur, up to five treatment shocks can be applied per treatment sequence by electrodes integrated in the garment. The time between the detection of an arrhythmia and the delivery of the treatment shock is generally less than 1 min. Recorded episodes as well as patient compliance reports are stored on a web‐based server (LifeVest Network) which can be accessed by the treating physician via personalized login data. Instead of inpatient monitoring with a manual defibrillation option, the WCD can effectively and systematically protect patients from SCD outside of the hospital.

The range of WCD use recommended by cardiology guidelines includes patients after ICD extraction, as well as for various primary and secondary prevention indications such as patients after myocardial infarction with an left ventricular ejection fraction (LVEF) dysfunction (≤35%), and patients with myocarditis or transient causes of LVEF dysfunction.^4^, ^5^, ^6^, ^7^

Despite proven safety and efficacy, there is still little data on the economic impact of WCD use, especially in patients following ICD explant due to infection. We therefore decided to perform a literature review to summarize the evidence related the efficacy, safety, and compliance of the WCD in patients after ICD explantation and to perform a cost‐minimization analysis to assess the economic impact associated with WCD use compared to the standard therapy.

## 2 METHODS

### 2.1 Literature search

The EUnetHTA health technology assessment (HTA) core model (EUnetHTA 2015) was used as a guideline for the literature search. Clinical, epidemiological, and economic aspects were considered.

Electronic databases (Medline, Pubmed, and Web of Science) were used for the literature search. Clinical and economic keywords related to ICD explantation, its treatment options, health outcomes, consequences for health‐related quality of life (HRQoL), and its economic implications were used. The search was performed using index/MeSH (Medical Subject Heading) and strings. Studies were selected based on the included indications, the age of the subjects, the year of publication (2008 and later) and the type of publication. We considered retrospective and prospective studies, randomized controlled trials, reviews, guidelines, and practice guides. Furthermore, studies on HRQoL in patients with sudden cardiac arrest (SCA) were used.

All publications were analyzed for efficacy, safety, and compliance in the target population, and duplicates were removed. Furthermore, all publications not containing information on the target population or the relevant aspects were excluded.

### 2.2 Cost minimization analysis overview

**FIGURE 1:**
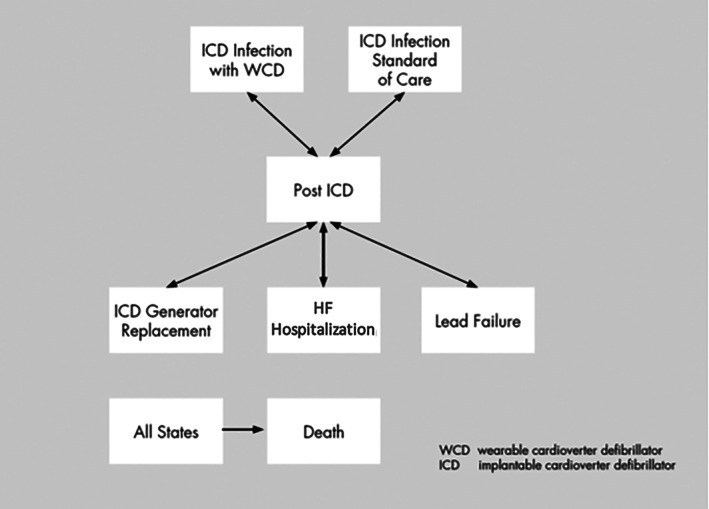
Markov model

We developed a decision‐analytical Markov model to simulate the long‐term clinical pathway and costs associated with the management of patients that required an ICD explantation due to infection. The model was used to perform a cost‐minimization analysis comparing two alternative treatment options: (1) WCD and (2) Standard of care in Italy, in order to understand the relative economic impact. (Figure 1) The decision to perform a cost‐minimization analysis was based on the conservative assumption that WCD and the standard of care in Italy had the same efficacy. The standard of care was hospitalization in a low‐intensity hospital after ICD explantation until the infection is cured and reimplantation is performed. Low‐intensity hospitals are hospitals dedicated to patients that have a lower risk compared to patients treated in intensive care, but liable to develop complications and in need of close monitoring much more than the standard care at home. They were established in Italy to meet the increasing need of long in‐hospital stay, which was previously managed in acute hospitals, and are used for patients that do not need an acute management.

The choice of a cost‐minimization analysis was made to perform a conservative analysis. The assumption of same efficacy, which is required to perform a cost‐minimization analysis instead of a cost‐effectiveness analysis, was considered conservative due to the evidence available that suggests a possible higher efficacy of WCD.^10^ Hospitalization in a low‐intensity hospital does not guarantee that patients are adequately protected from SCD unless they are in a monitor bed or on an intensive care unit.

To perform the cost‐minimization analysis, we retrieved data performing an extensive literature review. (Supplement Table 1) We discounted the costs at an annual rate of 3%.^12^, ^13^ The analyses were conducted from the perspective of the Italian National Health Service (NHS) and the results were presented in Euro (€). (Table 1).

**TABLE 1:** Base‐case scenario

Treatments	Cost	Cost discounted
WCD	€105 175.35	€86 035.52
Standard of care	€106 997.92	€87 817.92
	**∆ Cost**	**∆ Cost discounted**
WCD versus standard of care	−€1822.58	−€1782.40

*Note*: Δ, delta/difference.

Abbreviation: WCD, wearable cardioverter defibrillator.

### 2.3 Decision analytic model structure

We built a state‐transition Markov model using Microsoft Excel to assess the overall costs associated with the use of WCD, using a lifetime time horizon and monthly cycle. A hypothetical cohort of patients with ICD removed due to infection, with a mean age of 61 years (Supplementary Table 4) can receive the WCD after ICD removal or can be hospitalized in a low‐intensity hospital during the first monthly simulation cycle. Patients stay in this health state for 1 month; after this time period, we assumed the resolution of infection and the implantation of a new ICD. The new ICD implantation could be successful or result in procedural death. Patients with ICD entered in the post‐ICD health state, where they could be hospitalized for heart failure, experience new ICD infection, ICD generator replacement, and die from cardiac death or other causes.

### 2.4 Data input: Clinical data

Clinical data are reported in Supplementary Table 4. Based on the conservative approach of the cost‐minimization analysis, we assumed a comparable WCD effectiveness to the standard therapy in Italy. The effectiveness of the intervention was presented based on the incidence of SCA in the first month after ICD extraction and WCD event survival rates of 85.5%. Event survival rate was defined as SCA rate due to VT/VF events in the context of all SCA events including, for example, asystole, with a 100% termination‐success rate of VT/VF events by the WCD.^10^, ^14^ Cardiac and noncardiac deaths were presented and analyzed separately for the assessment of long‐term mortality after ICD reimplantation, in accordance with Woo et al.^15^ Furthermore, the mortality risk was split and separately assessed according to general (noncardiac) causes of death, which increase with age, and specific mortality risks, which are particularly present in patients with heart disease. Data on general population mortality were taken from the Italian Institute of Statistics (ISTAT).

In order to assess excess mortality in patients with ICDs, total mortality in the ICD group and the prevalence of SCD were estimated from the SCD‐HeFT (Sudden Cardiac Death in Heart Failure) and MADIT‐II (Multicenter Automatic Defibrillator Implantation Trail) studies.^16^, ^17^ During the simulation of the model, excess cardiac mortality remained constant, allowing the mortality of the general population to increase with age. Thus, the proportion of SCD in the overall mortality risk decreased over time.

Furthermore, the mortality risk associated with ICD implantation and ICD side effects was integrated into the model.^18^, ^19^, ^20^ The probabilities of lead failure or ICD infection were estimated based on published registry data.^18^, ^19^, ^20^, ^21^, ^22^, ^23^

### 2.5 Data input: Costs

Cost data are reported in Supplementary Table 4. For the simulation 30 days after ICD explant, we considered WCD costs, ICD reimplantation costs, as well as costs for inpatient stay, here considered the standard therapy. WCD costs were estimated to be €3600, according to the Italian average price (provided by ZOLL Medical Italia srl), and the cost of inpatient therapy in a low‐intensity hospital was estimated to be €5250 (€250 daily hospital costs and 21 days hospital stay). Costs for ICD implantation, as well as costs for possible subsequent complications were taken from the Italian DRG system. Accordingly, the costs of HF hospital stay for ICD patients were calculated. A mean ICD battery life of 5 years was assumed, excluding the possibility of battery failure within the first 2 years.^24^, ^25^ The mean monthly cost of a patient after ICD implantation was determined based on the results of Smith et al.^26^

### 2.6 Analysis

We conducted a base case analysis to assess the costs of WCD therapy and standard of care, as well as the difference in total costs associated with these interventions.

We also performed a sensitivity analysis assessing the impact of WCD and hospitalization cost reducing and or increasing the parameter from −30% to +30%. This analysis provides valuable information to understand the impact of treatment cost on the results and gives the possibility to understand the economic impact in scenarios where WCD and standard of care have different costs compared to what was assumed in our base case analysis. The hospitalization cost of €5250, used in our base case analysis, was estimated by multiplying the daily cost of hospitalization (€250) by 21 days of hospital length of stay. Based on this cost estimation, assessing a reduction of −30% of hospitalization cost means reducing the day cost of 30% (from €250 to €175 per day) or reducing the length of stay from 21 to 15 days. Same meaning is associated to increasing the hospitalization cost by 30% (from €250 to €325 per day or hospital length of stay from 21 to 27 days).

Finally, a one‐way sensitivity analysis was carried out to confirm the reliability of the results and to determine the influence of the individual parameters.

Since our research was based on a systematic review of published literature, with no direct patient involvement, ethical approval was not required and patient consent was not applicable.

## 3 RESULTS

### 3.1 WCD efficacy, safety, and compliance

Twenty‐six original studies were analyzed to evaluate the efficacy, safety, and compliance of the WCD in patients after ICD explantation. A total of 14 studies were included in the analyses, including our target population explanted ICD. Thirteen studies were retrospective, and two studies were prospective. (Suppl. Table S1).^8^, ^11^, ^14^, ^27^, ^28^, ^29^, ^30^, ^31^, ^32^, ^33^, ^34^, ^35^, ^36^, ^37^, ^38^ Three studies had an exclusive focus on patients after ICD removal.^8^, ^11^, ^31^ In addition, the only available RCT for WCD use was consulted to verify the results of the registry data.^39^ In some studies with a mixed patient population, no specific results could be determined for our population. Overlaps between the studies were excluded. All included studies considered effectiveness, safety, and compliance. The comparison of the studies' results is difficult due to differences in design and observation periods. (Supplement Tables 2–3) However, it can be concluded for all evaluated studies that the WCD is able to protect patients safely and effectively from SCD after ICD removal. The rate of inappropriate shocks was extremely low (<0.6%) in all studies.^8^, ^11^, ^14^, ^27^, ^28^, ^29^, ^30^, ^31^, ^32^, ^33^, ^34^, ^35^, ^36^, ^37^, ^38^, ^40^ In almost all evaluated studies, patients demonstrated a compliance >20 h per day. Also the as‐treated analysis of the VEST trial^41^ stressed how compliance to WCD, in terms of hours in a day actually wearing the device, is a key factor in conditioning the effect of this intervention on outcomes and this implies that patient education and selection are crucial.

According to Tanawuttiwat et al., mortality is 8.2% in patients after device removal due to infection. The authors described the WCD as useful in protecting patients in the bridging period after removal until reimplantation.^8^ Ellenbogen et al. concluded that the WCD provides physicians with more flexibility in their treatment of patients after ICD explantation by protecting them during the high‐risk period, and by allowing time for the determination of a long‐term risk management strategy.^11^

### 3.2 WCD cost‐minimization in patients after ICD explantation due to infection in Italy

**FIGURE 2:**
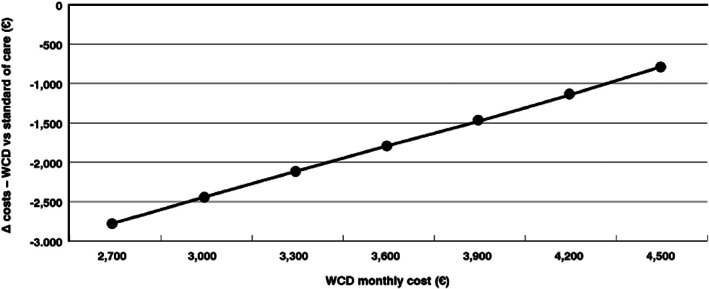
WCD cost‐minimization in relation to WCD cost. Variation of WCD cost with stable standard of care costs. The higher the WCD costs, the lower the difference to the costs of standard therapy and vice versa. Within the known renting costs range and further, there will be savings with the WCD approach. WCD, wearable cardioverter defibrillator

**FIGURE 3:**
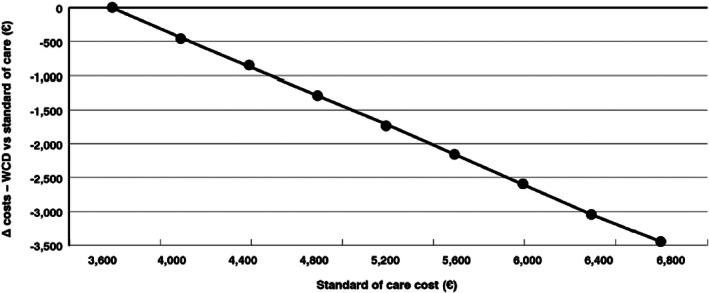
WCD cost‐minimization in relation to standard of care cost. Variation of standard of care costs with stable WCD costs. The higher the standard of care costs, the greater the savings with the WCD approach. WCD, wearable cardioverter defibrillator

We conducted a cost‐minimization analysis for the WCD in comparison to the standard therapy (low‐intensity inpatient hospitalization). In the basic scenario, WCD therapy proved not only to be cost‐effective, but cost saving. Cost savings of €1782 per patient were gained when using the WCD (Table 1). Both the costs of the WCD and the costs of standard therapy influenced the results. (Figures 2 and 3) In the Figure 2, we assessed the impact of different WCD prices on the possible cost savings associated to WCD. Assuming WCD costs ranging from €2700 to €4500, the WCD remained cost saving with a cost reduction of €2800 using a WCD price of €2700 and of €810 using a price of €4500. The same analysis was performed, modifying the standard of care costs (Figure 3). In this analysis, the WCD costs were fixed. The WCD presents here as well with cost savings, even if we reduced the standard of care costs to €3600. (Figure 3) When increasing the standard of care costs to €6800, cost savings of €3500 were associated to WCD. In the one‐sided‐sensitivity analysis, WCD costs and standard therapy were confirmed to be the main influencing factors on the results of the cost minimization analysis. (Supplementary Figure 1).

## 4 DISCUSSION

The proper care of patients after infection‐related ICD removal is a challenge for many reasons. The substantial and persistent risk for SCD is already confirmed in all ICD patients. After ICD removal, the patient is unprotected from their risk of SCD, due to the respective underlying disease. This risk even increases from the infection and explant procedure itself. The consequences of a survived, but inadequately treated SCA can lead to considerable costs and impairments both on an economic and patient level.^10^ These costs could increase considerably depending on the time needed for adequate therapy, that is, defibrillation.^42^

Life‐threatening arrhythmias usually occur unexpectedly, unobserved, often at home and during sleep.^43^ The initial probability of survival is less than 7% due to delayed defibrillation or no defibrillation at all.^27^, ^44^, ^45^ The 30‐day survival rate is only 2.4%.^46^ Up to 50% of SCA survivors cope with serious consequences such as long‐term severe neurological damage, cognitive impairment, depression, and post‐traumatic stress disorder.^47^, ^48^, ^49^, ^50^, ^51^ An Italian pilot study on early defibrillation by volunteers using publicly available AEDs was able to reduce the SCD rate, but not to the expected or desired extent.^52^ This was due to the fact that most high‐risk patients spend an insufficient amount of the day in public places, and SCA is more likely to occur at home.^51^

The costs incurred by SCA/SCD also represent a considerable burden on the country‐specific health care system. Weng et al. estimate the costs at discharge from hospital at $32 000, with subsequent costs of $12 953 in the first year after SCA.^53^ However, the costs and outcomes vary depending on the time of defibrillation. Van Alem et al. calculated that in the case of defibrillation after 2 min, the probability of survival is about 46% with costs around $20 253. Remarkably, if defibrillation is performed just after 6 min, the probability of survival is as low as 13%, resulting in costs around €27 781.^42^ The earlier a patient is defibrillated, the higher the probability of patient survival and the lower the associated costs. This, unfortunately, presents a conflict with the general response times of emergency medical systems (EMS), which vary between 10 and 15 min on average in Europe.^44^

The back‐up defibrillation therapy ensured by an ICD is the gold standard in patients with a confirmed long‐term increased risk of SCD. If an ICD infection occurs, explantation of the device, the leads, or both is often unavoidable, as the infection is associated with significant mortality, morbidity and costs. The mortality rate is around 8–26%.^8^ The number of hospital admissions caused by cardiac device‐related infections rose from 5308 in 2003 to 9948 in 2011, and associated costs also increased from $91 348 to $173 211, accordingly.^9^ The management of patients after ICD explantation is difficult, as the patients are unprotected from their predetermined high risk of SCD.^3^, ^54^, ^55^ Alternatives for protecting these patients are limited. The risk of mortality due to device infection only adds to the preexisting and predetermined risk of SCD. After explantation, patients have a 4–6% risk of experiencing a life‐threatening VT/VF event.^8^, ^11^ Apart from a WCD, there are hardly any alternatives available that adequately protect the patient, and are economically attractive at the same time. Inpatient monitoring is not a feasible alternative. Early ICD reimplantation is not recommended by the guidelines due to the high risk of reinfection. The WCD, as a noninvasive external cardioverter defibrillator, has been established in clinical routine for various indications in over a decade. It effectively covers the bridging time from hospital discharge until possible device reimplantation. Efficacy, safety, and compliance of the WCD has been confirmed in several indications by various retro‐ and prospective registry data as well as in an RCT. In conclusion, the WCD is a useful bridging tool to reimplantation, protecting patients at risk from SCD by delivering a timely and reliable defibrillation if and when needed. In explanted patients, the clinical value of the WCD is not only demonstrated by the number of terminated or avoided arrhythmic events, but also by allowing for protected risk assessment outside of the hospital, and the possibility to perform guideline‐based reimplantations. The WCD is therefore recommended by national and international cardiology societies for various indications, especially for use in patients after ICD removal due to infection.^4^, ^5^, ^6^, ^7^

To better assess the economic significance of WCD use, three studies published between 2015 and 2017 were analyzed. Two publications included mixed patient populations. One study focused on patients after ICD removal. In summary, all studies showed positive and cost‐effective WCD use, although the studies differed in method, design, investigated collective and time horizon, setting and type of analysis.

Only few studies have been published to date which focus on the economic implications of the WCD.

In the largest study, Healy and Carrillo developed a Markov model for the US healthcare system to demonstrate cost‐effectiveness of WCD use in patients after ICD removal.^10^ They analyzed direct costs (e.g., cost of WCD device, hospital costs, cost of laboratory tests, cost of follow‐up visits, and costs related to ICD implantation and management) as well as indirect costs (loss of income and loss of productivity for premature death). The starting point of the investigations was the assumption of four possible patient management options: discharge home with or without WCD, discharge to a skilled nursing facility without WCD and further inpatient monitoring. The quality adjusted life years (QALYs) and life year (LYs) gained were calculated as parameters of effectiveness between the alternative strategies. According to their calculations, the Incremental Cost‐Effectiveness Ratio (ICER) of the WCD strategy as compared to unprotected patient discharge from hospital amounted to $20 300 per LY and $26 436 per QALY gained. In comparison to the other alternatives, the WCD proved again to be cost effective. In fact, patient discharge to a skilled nursing facility and in‐hospital monitoring resulted in higher costs and worse clinical outcomes. Healy and Carrillo performed a one‐ and two‐sided sensitivity analysis to reflect result uncertainties. The SCA event rate, WCD treatment efficacy, and time to reimplantation had the greatest influence on the ICER. Overall, WCD cost‐effectiveness decreased with declining SCA event rate. WCD cost‐effectiveness increased with higher WCD efficacy. If WCD efficacy of 95% or <69% was considered, the ICER was between $15 392/QALY and >$50 000/QALY. Assuming a SCA risk of 5.6% over a two‐month period, the WCD remained cost‐effective as long as the time to reimplantation was at least 2 weeks.^10^

We conducted a cost‐minimization analysis to further investigate these statements. In this analysis, the WCD demonstrated cost savings of €1782 per patient, compared to the comparative therapy, further inpatient stay. These calculations were based on the assumption of equivalent WCD effectiveness with the standard of care (three weeks hospital stay in a low‐intensity hospital). Considering the results of Healy and Carillo,^10^ who estimated the effectiveness of the WCD to be higher than standard therapy, our assumptions are rather conservative, and the resulting calculations are likely an underestimation of the potential savings. However, even with this conservative approach, the WCD proves to be cost saving for the Italian NHS budget. These results were confirmed in a sensitivity analysis, and are most likely applicable to other healthcare systems internationally as well. Even by significantly changing the WCD costs, the analysis reported the WCD as a cost saving option with a range from −€2800 (using WCD costs of €2700) to −€810 (using WCD costs of €4500).

Some limitations must be considered when reviewing the present analysis. The main limitation of this analysis is connected with the absence of a direct comparison provided by a randomized controlled trial. As there was no direct comparison between the effectiveness of a WCD approach with in‐hospital management at hand, we assumed equal effectiveness, despite the fact that in a normal ward manual defibrillation within few minutes cannot be guaranteed 24/7, especially during sleep of a patient, and therefore may be inferior to WCD use. However, a specific cost‐effectiveness analysis based on trial data is needed to estimate a more precise value of the WCD in this setting. The considered standard of care and costs were taken from the specific Italian situation and may be adapted to other health care systems.

Nevertheless, our cost‐minimization analysis may help decision‐makers to better understand not only the clinical, but also the potential economic value of the WCD in patients after infection‐related ICD removal.

## 5 CONCLUSION

The use of WCD for protecting patients at risk of SCD who require ICD explantation is a safe and effective strategy for SCD protection, as well as cost saving. The cost‐minimization analysis demonstrated a cost reduction of €1782 per patient using the WCD. The WCD allows for a flexible and individualized treatment of patients after ICD explant. Furthermore, it provides physicians with the needed time to develop a guideline directed long‐term risk management strategy for their patients. The need for such temporary protection is justified by the high rate of life‐threatening arrhythmias caused by the underlying disease, the infection, and the explant procedure itself. Our analysis supports the few limited findings so far regarding the economic impact of WCD. Additional studies may follow to further substantiate the cost‐effectiveness and cost saving potential of the WCD. For now, however, the use of WCD in patients undergoing ICD removal is reasonable from a clinical and economic perspective in the Italian NHS, and quite possibly in other national health care systems as well.

## CONFLICT OF INTEREST

Lorenzo Giovanni Mantovani has received grants and personal fees from Bayer AG, Boehringer Ingelheim, Pfizer, and Daiichi‐Sankyo. Paolo Angelo Cortesi received a research grant from Baxalta now part of Shire, and speaking honoraria from Pfizer and Roche. Giovanni Luca Botto: speaker fees (modest amount) from Abbott, Biotronik, Boston Scientific, Medtronic, Zoll. Roberto De Ponti: speaker fees (modest amount) from Biosense Webster and Biotronik. Federico Guerra: speaker fees (modest amount) from Boston Scientific, Medtronic, Zoll.

## Supporting information


**Appendix** S1: Supporting information

## Data Availability

The data that supports the findings of this study are available in the supplementary material of this article.
