# Supplementary material for: Cost‐minimization analysis of a wearable cardioverter defibrillator in adult patients undergoing ICD explant procedures: Clinical and economic implications
Source: Clin Cardiol. 2021 Aug 24;44(11):1497–505. doi: 10.1002/clc.23709 (PMC8571546; doi:10.1002/clc.23709)

**Supplementary files**

Table 1

Table 2

Table 3

Table 4. Clinical data input

| **Parameter** | **Value** | **References** |
| --- | --- | --- |
| Cohort mean age | 61 | Healy 2015 |
| Discount rate - cost | 0.030 | Sanders 2016, Cortesi 2018 |
| *30 days after ICD explanation* | | |
| Mortality WCD probability | 0.0404  (0.0325 - 0.0484) | Chung 2010, Healy 2015 |
| Mortality Standard of Care | 0.0404  (0.0325 - 0.0484) | Chung 2010, Healy 2015; assumption |
| *ICD* | | |
| Procedural death - monthly probability | 0.0020  (0.0016-0.0024) | van Rees 2014 |
| Cardiac mortality in patients with ICD - monthly probability | 0.0044  (0.0035-0.0052) | Bardy 2005; Greenberg 2004 |
| Probability of lead failure - monthly probability | 0.0015  (0.0012-0.0018) | Kremers 2013 |
| Probability of ICD infection - monthly probability | 0.0007  (0.0006-0.008) | Kremers 2013, Uslan 2007, Margery 2010, Johansen 2011 |
| Death from lead failure - monthly probability | 0.0130  (0.0104-0.0156) | Cheng 2010 |
| Battery life (replacement) | 0.0113  (0.0091-0.0136) | Kramer 2013b; Gandjour 2011 |
| Hospitalization for HF | 0.0080  (0.0064-0.0096) | Moss 2009; Tang 2010 |

Table 5. Cost data inputs

| **Parameter** | **Value** | **References** |
| --- | --- | --- |
| *Costs* | | |
| Cost of WCD (1 month) | 3,600 €  (2,894 €-4,306 €) | Assumption |
| Cost of low intensity care (21 days) | 5,250 €  (4,221 €-6,279 €) | ASST rhodense, Expert opinion |
| Cost of ICD explanation due to non fatal infection | 21,634 €  (17,394 €-25,874 € | DRG 536 – G.U. 2013 |
| 30 days after ICD infection - WCD CD | 25,234 €  (20,288 €-30,180 €) | Assumption, DRG 536 – G.U. 2013 |
| 30 days after ICD infection – Standard of care | 26,884 €  (21,615 €-32,153 €) | ASST rhodense, Expert opinion, DRG 536 – G.U. 2013 |
| Cost of ICD reimplantation after non fatal infection | 0 € | - |
| Cost of ICD replacement | 16,573 €  (13,258 €-19,888 €) | DRG 515 – G.U. 2013 |
| Cost of revise due to lead problem | 3,547 €  (2,837 €-4,256 €) | DRG 117 – G.U. 2013 |
| Monthly cost after ICD implantation | 345 €  (149 €-541€) | Madotto 2015 |
| HF hospitalization costs (average cost for an HF hospitalization) | 4,898 €  (3,918 €-5878 €) | D'angiolella 2019 |

## **Figure 1.** Tornado Diagram – one way sensitivity analysis


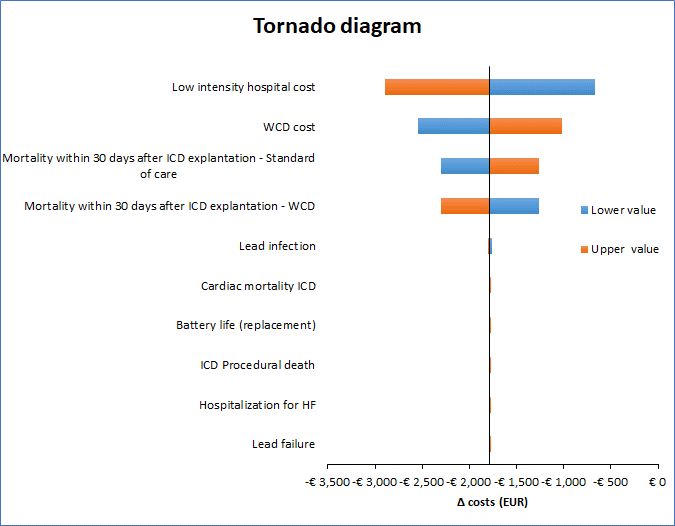

Supplement: Supplementary file 1 — Appendix S1: Supporting information [file CLC-44-1497-s001.docx]
